# Phase Regulation and Defect Passivation Enabled by Phosphoryl Chloride Molecules for Efficient Quasi-2D Perovskite Light-Emitting Diodes

**DOI:** 10.1007/s40820-023-01089-3

**Published:** 2023-05-01

**Authors:** Mingliang Li, Yaping Zhao, Jia Guo, Xiangqian Qin, Qin Zhang, Chengbo Tian, Peng Xu, Yuqing Li, Wanjia Tian, Xiaojia Zheng, Guichuan Xing, Wen-Hua Zhang, Zhanhua Wei

**Affiliations:** 1 Sichuan Research Center of New Materials, Institute of Chemical Materials, China Academy of Engineering Physics, Chengdu, 610200 People’s Republic of China; 2 Xiamen Key Laboratory of Optoelectronic Materials and Advanced Manufacturing, Institute of Luminescent Materials and Information Displays, College of Materials Science and Engineering, Huaqiao University, Xiamen, 361021 People’s Republic of China; 3 Joint Key Laboratory of the Ministry of Education, Institute of Applied Physics and Materials Engineering, University of Macau, Taipa, Macau, 999078 People’s Republic of China; 4 Innovation Laboratory for Sciences and Technologies of Energy Materials of Fujian Province (IKKEM), Xiamen, 361005 People’s Republic of China; 5 School of Materials and Energy, Yunnan University, Kunming, 650050 People’s Republic of China

**Keywords:** Quasi-2D perovskite, Phosphoryl chloride functional group, Crystallization control, *N* phase control, Passivation

## Abstract

**Highlights:**

- The modification of perovskite precursor by a series of phosphoryl chloride molecules can indeed improve the performance of perovskite LEDs (Pero-LEDs).
- The bis(2-oxo-3-oxazolidinyl) phosphinic chloride can not only regulate the phase distribution by controlling the crystallization rate but also passivate the defects of the quasi-2D perovskite.
- Highly efficient and reproducible Pero-LEDs are achieved with an maximum external quantum efficiency (EQE_max_) of 20.82% and an average EQE (EQE_ave_) of around 20% on 50 devices.

**Abstract:**

Quasi-2D perovskites have attracted tremendous interest for application as light-emission layers in light-emitting diodes (LEDs). However, the heterogeneous n phase and non-uniform distribution still severely limit the further development of quasi-2D perovskite LEDs (Pero-LEDs). Meanwhile, the increased defect density caused by the reduced dimension and grain size induces non-radiative recombination and further deteriorates the device performance. Here, we found that a series of molecules containing phosphoryl chloride functional groups have noticeable enhancement effects on the device performance of quasi-2D Pero-LEDs. Then, we studied the modification mechanism by focusing on the bis(2-oxo-3-oxazolidinyl) phosphinic chloride (BOPCl). It is concluded that the BOPCl can not only regulate the phase distribution by decreasing the crystallization rate but also remain in the grain boundaries and passivate the defects. As a result, the corresponding quasi-2D Pero-LEDs obtained a maximum external quantum efficiency (EQE_max_) of 20.82% and an average EQE (EQE_ave_) of around 20% on the optimal 50 devices, proving excellent reproducibility. Our work provides a new selection of molecular types for regulating the crystallization and passivating the defects of quasi-2D perovskite films.
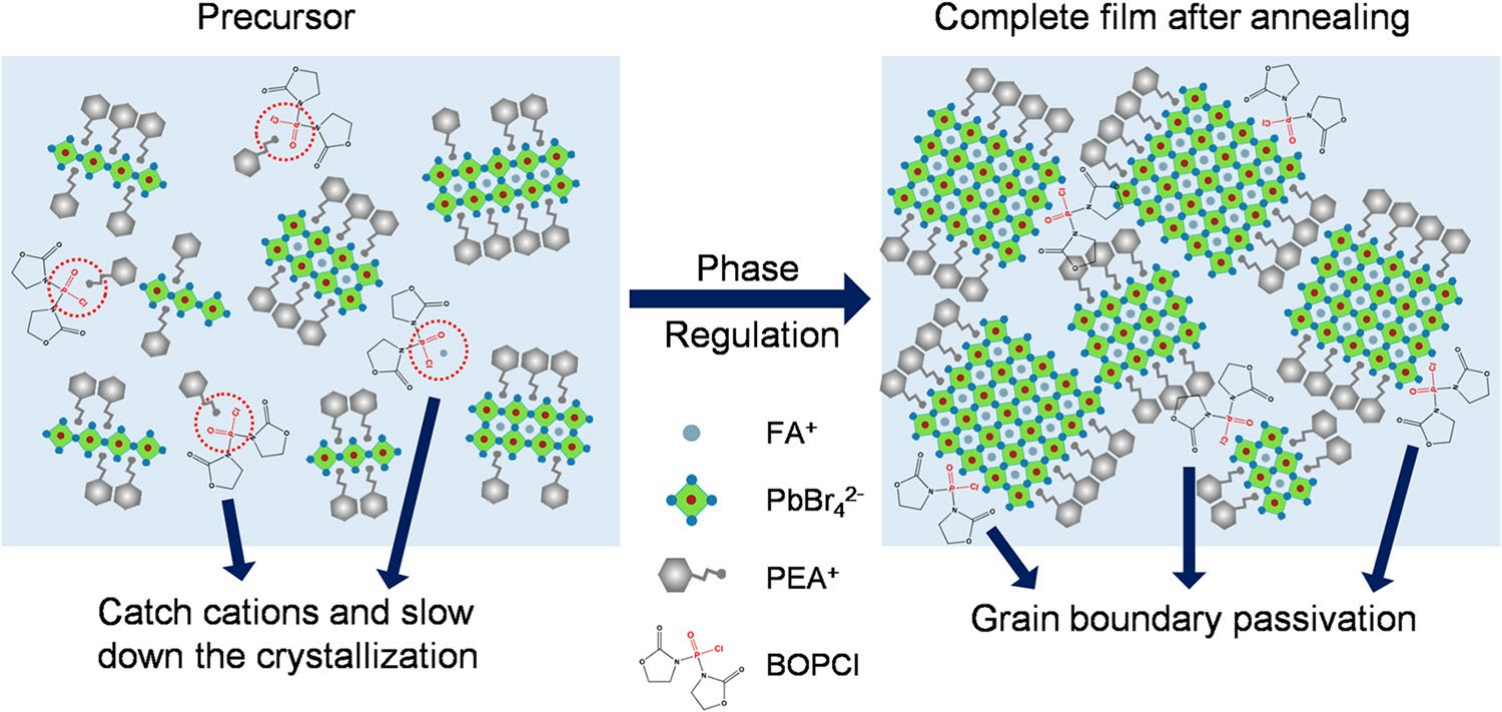

**Supplementary Information:**

The online version contains supplementary material available at 10.1007/s40820-023-01089-3.

## Introduction

Recently, metal halide perovskites are emerging as superb semiconductors for light-emitting diodes (LEDs) due to their excellent optoelectronic characteristics such as high color purity, adjustable bandgap and high defect tolerance [1–4]. External quantum efficiencies (EQEs) of perovskite LEDs (Pero-LEDs) have been greatly improved in the past few years. The EQEs of green, red and near-infrared Pero-LEDs have exceeded 20%, and the blue ones have also exceeded 18% [5–9]. Recently, quasi-2D perovskites have been intensely investigated as the emission layers in the Pero-LEDs due to their unique multi-quantum-well structure, providing a funneling channel for carriers and promoting radiative recombination [10–13]. However, there remain some challenges limiting the device performance. For example, the rapid crystallization process and low formation energy result in the heterogeneous distribution of various-*n* phases, especially the excessive low-*n* (*n* ≤ 2) phases. This complicated phase distribution would result in low carrier injection and energy transfer efficiency, severely restricting the further development of quasi-2D Pero-LEDs [14–16]. Meanwhile, an increased defect density caused by the reduced dimension and grain size of quasi-2D perovskites would also promote the non-radiative recombination and further deteriorate the photoelectric performance of quasi-2D Pero-LEDs [17–19].

A good deal of effort has been taken to control the n phases distribution of quasi-2D perovskites and passivate the defects caused by the reduced dimension and grain size [10, 11, 14–16, 20]. Guo et al. used a series of alkali metal bromides to control the nucleation and growth [10]. They found that adding potassium ions could limit the nucleation of high-*n* phases (*n* ≥ 3) and allow the subsequent growth of low-*n* phases, which helps different *n* phases to be distributed more evenly and promote energy transfer. Ma et al. applied fluorinated triphenylphosphine oxide (TFPPO) to control the diffusion of organic cations and suppress the formation of low-*n* phases [11]. Meanwhile, the TFPPO could also passivate the perovskite grain boundaries. Yu et al. introduced potassium tetrafluoroborate (KBF_4_) to finely regulate the phase distribution and reduce the defect density of quasi-2D perovskite [14]. The introduction of BF_4_^−^ ion significantly inhibits the nucleation of low-*n* phases and induces the nucleation of high-*n* phases. Moreover, the BF_4_^−^ ion could also fill the Br^−^ anion vacancy of quasi-2D perovskite to reduce the non-radiative recombination. Although a number of studies on phase control and defect passivation have been reported, there are very few reports on the limitation of component diffusion and crystallization delay of quasi-2D perovskites.

Here, we found that a series of molecules containing phosphoryl chloride functional groups, when added to the perovskite precursor, have noticeable enhancement effects on the device performance of quasi-2D Pero-LEDs. Then, we studied the modification mechanism by focusing on the bis(2-oxo-3-oxazolidinyl) phosphinic chloride (BOPCl) and found that the BOPCl can slow down the crystallization rate of quasi-2D perovskite films, thus inhibiting the mass formation of low-*n* phases and promoting the growth of high-*n* phases. Moreover, the BOPCl molecules would remain in the grain boundaries, interact with the uncoordinated cations and passivate the non-radiative defects. Finally, the champion quasi-2D Pero-LEDs with the addition of BOPCl have obtained a maximum EQE (EQE_max_) of 20.82%, and the average EQE (EQE_ave_) of the 50 best components is about 20%, showing good reproducibility. Our work provides a new basis for the selection of molecular types for the crystal regulation and defect passivation of quasi-2D perovskite films.

## Experimental Section

### Materials

Phenylethylammonium bromide (PEABr), formamidinium bromide (FABr) and methylammonium chloride (MACl) were purchased from Greatcell Solar. BOPCl, bis(dimethylamino)phosphoryl chloride (BDPCl), dimethyl chlorophosphate (DClP), diphenyl chlorophosphite (DOPCl) and diphenylphosphinic chloride (DPCl) were purchased from Tokyo Chemical Industry Co., Ltd. Tris[2,4,6-trimethyl-3-(pyridin-3-yl)phenyl]borane (3TPYMB) was acquired from Xi’an Polymer Technology Crop. Except for the chemicals mentioned above, all the others were purchased from Sigma-Aldrich. All the materials were used as received without any purification.

### Preparation of Perovskite Precursor

PEABr, PbBr_2_, FABr and MACl were dissolved in dimethyl sulfoxide (DMSO) with a molar ratio of 2:3:2:0.3 to form the Control-Pero precursor. The concentration for PbBr_2_ was 0.5 M. And the BOPCl-Pero precursor was prepared by adding 5% BOPCl (as the molar ratio to PbBr_2_) into the Control-Pero precursor. Samples modified with other phosphoryl chloride molecules were also prepared by directly adding the corresponding molecules into the precursor. The corresponding addition ratio was BDPCl-7%, DClP-10%, DOPCl-13% and DPCl-15% to PbBr_2_.

### Device Fabrication

The patterned indium tin oxide (ITO) conductive glass substrates were ultrasonically cleaned in detergent solution, deionized water, acetone, isopropyl alcohol and ethanol and then dried with compressed nitrogen (N_2_). The substrates were further treated with UV-Ozone for 30 min before use. Then, the ITO substrates were put on a hot plate for spray pyrolysis deposition. The hot plate started programmed heating from room temperature to 530 °C within 20 min. Meanwhile, 5 mg of lithium acetate, 32 mg of magnesium acetate and 200 mg of nickel acetylacetonate were dissolved in a mixture of 45 mL of acetonitrile and 10 mL of ethyl alcohol to form a NiMgLiO_*x*_ precursor. When the temperature reached 530 °C, the NiMgLiO_*x*_ precursor was sprayed and pyrolyzed on the ITO substrates, forming a layer of NiMgLiO_x_. Compressed air was used as the working gas. The whole spraying process lasted about 10 min. After spraying, the substrates were kept heating at 530 °C for 20 min. After cooling to room temperature, the substrates were transferred into a N_2_-filled glovebox. For the perovskite emitting layers, 60 μL of perovskite precursor solution was spin-coated onto the ITO/NiMgLiO_*x*_ substrates at 4000 rpm for 60 s, and 300 uL of chlorobenzene (CB) was added as antisolvent at 20 s. Then, the perovskite films were annealed at 100 °C for 5 min. For passivation, 100 µL 1.5 mg mL^−1^ trioctylphosphine oxide (TOPO) in CB was spin-coated on the perovskite films at 4000 rpm for 1 min, and the perovskite films were annealed at 100 °C for 5 min again. Finally, a 70-nm-thick layer of 3TPYMB, a 1-nm-thick layer of lithium fluoride (LiF) and a 120-nm-thick layer of aluminum (Al) were deposited using a thermal evaporation system under a vacuum of < 1.0 × 10^–4^ Pa. The active area was 3 mm^2^ (2 mm × 1.5 mm).

### Characterization

All devices were measured in the N_2_-filled glovebox using a Keithley 2450 instrument and a QE-Pro spectrometer. X-ray photoelectron spectroscopy (XPS) and ultraviolet photoelectron spectroscopy (UPS) were obtained on an expanding X-ray Photoelectron Spectrometer (Thermo Fisher ESCALAB Xi +). UV–Vis absorption, steady-state photoluminescence (PL) spectra and photoluminescence quantum efficiency (PLQY) were obtained using a QE-Pro spectrometer in the glovebox. Time-resolved photoluminescence (TRPL) curves were measured by a Fluorescence spectrophotometer (Edinburgh FLS920) with a pulsed excitation laser of 375 nm. Fourier-transform infrared (FTIR) spectroscopy measurement was conducted on a Nicolet IS50 FTIR spectrometer (Thermo Scientific). Scanning electron microscopy (SEM) was carried out on a field-emission scanning electron microscope (JEOL JSM-7610F). Atomic force microscopy (AFM) was measured using the Bruker Multimode 8 AFM instrument. Grazing incidence wide-angle X-ray scattering (GIWAXS) measurement was performed at the Xenocs (Xeuss) with a photon wavelength of 1.5418 Å. The detector was carried out a PILAUTS3 R 300 K area and placed 130 mm from the sample center. X-ray diffraction (XRD) patterns were obtained from a SmartLab X-ray diffractometer (Rigaku Corporation) with a Cu κα radiation source. Transient absorption (TA) spectroscopy was performed using the Ultrafast System HELIOS TA spectrometer, where the pump wavelength was 365 nm generated from a Light Conversion TOPAS-C optical parametric amplifier. The corresponding laser source was from a Coherent Legend regenerative amplifier (150 fs, 1 kHz, 800 nm), seeded by a Coherent Vitesse oscillator (100 fs, 80 MHz). And the broadband probe pulses (420–780 nm) were generated by focusing a small portion (≈10 μJ) of the primary 800 nm laser pulses into a 2-mm sapphire plate.

## Results and Discussion

### Device Performances

**Fig. 1:**
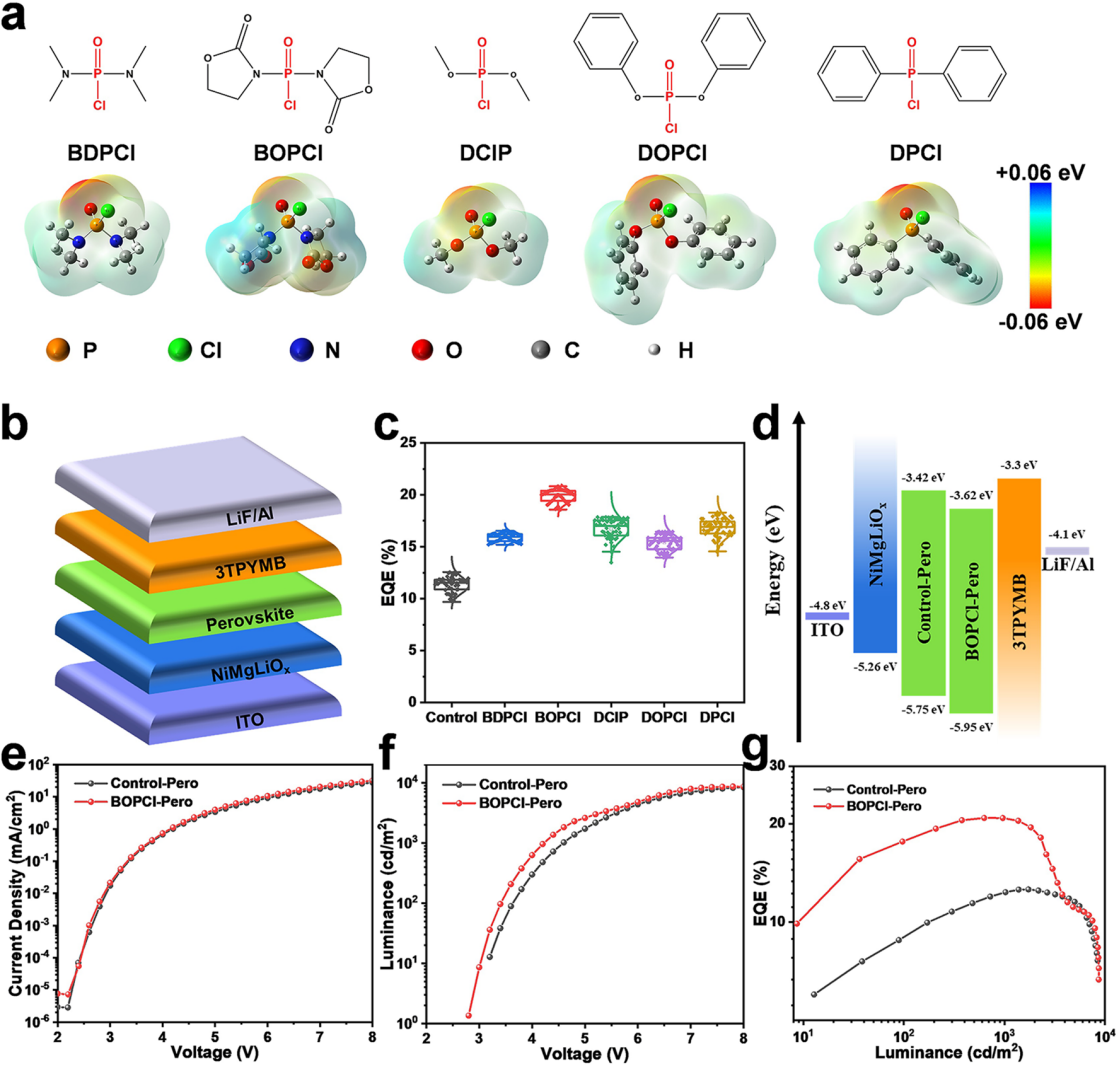
**a** Molecular structures and electrostatic potentials of the BDPCl, BOPCl, DClP, DOPCl and DPCl. **b** Device structure of the Pero-LEDs. **c** EQE_max_ histogram of the best 50 devices based on Control-Pero films and other perovskite films modified by various phosphoryl chloride molecules. **d** Energy-level diagram, **e** current density–voltage, **f** luminance–voltage and **g** EQE–luminance curves of Pero-LEDs based on Control-Pero and BOPCl-Pero films

The molecular structures and electrostatic potentials of the phosphoryl chloride molecules we used are shown in Fig. 1a, including BDPCl, BOPCl, DClP, DOPCl and DPCl. The phosphoryl chloride functional groups are highlighted in red, and the electrostatic potential of these phosphoryl chloride molecules proves that the negative potential mainly populates at their oxygen/chlorine atoms. As shown in Fig. 1b, we fabricated the quasi-2D Pero-LEDs with the device structure of ITO/NiMgLiO_*x*_/perovskite/3TPYMB/LiF/Al, and the corresponding cross-sectional SEM image of the Pero-LEDs is shown in Fig. S1. We first made statistics for EQE_max_ on the 50 devices based on Control-Pero films and other perovskite films modified with these phosphoryl chloride molecules, as shown in Fig. 1c, and the corresponding maximum current efficiencies (CE_max_) are demonstrated in Fig. S2. It can be found that the Pero-LEDs based on the films modified with these phosphoryl chloride molecules had better performance than the Control-Pero films, indicating that the modification of phosphoryl chloride molecules can indeed improve the performance of Pero-LEDs. Among these molecules, the BOPCl showed the best modification effect, with an EQE_ave_ of around 20%, showing excellent reproducibility.

In order to study the modification mechanism of these phosphoryl chloride molecules, we focused on the BOPCl for further exploration. The as-prepared perovskite films’ energy levels were determined using the UPS results (Fig. S3). And the whole device energy-level diagram is exhibited in Fig. 1d. Compared with the Control-Pero film, the BOPCl-Pero film had a lower valence band energy level of − 5.95 eV. As displayed in Fig. 1e, the current density of the Pero-LEDs based on the two films did not change significantly, implying that the added BOPCl did not bring in side effect on charge injection and transport, while, at low voltage (3–6 V), the luminance of the BOPCl-modified device increased nearly twice at almost the same current density in Fig. 1f, indicating that the radiation recombination of the perovskite film was significantly enhanced after the BOPCl modification. Thus, the EQE of the BOPCl-Pero device was nearly doubled (Fig. 1g). After BOPCl modification, the EQE of the device increased from 12.8 to 20.8% compared with the control sample. Electroluminescence (EL) spectra of the Pero-LEDs based on Control-Pero and BOPCl-Pere films are shown in Fig. S4. The almost identical luminescent peak position certified that the introduction of BOPCl did not change the perovskite luminescence center. The CIE coordinate of Pero-LEDs based on the BOPCl-Pero film is exhibited in Fig. S5, presenting a pure green emission.

### Analysis of Film Forming Process

**Fig. 2:**
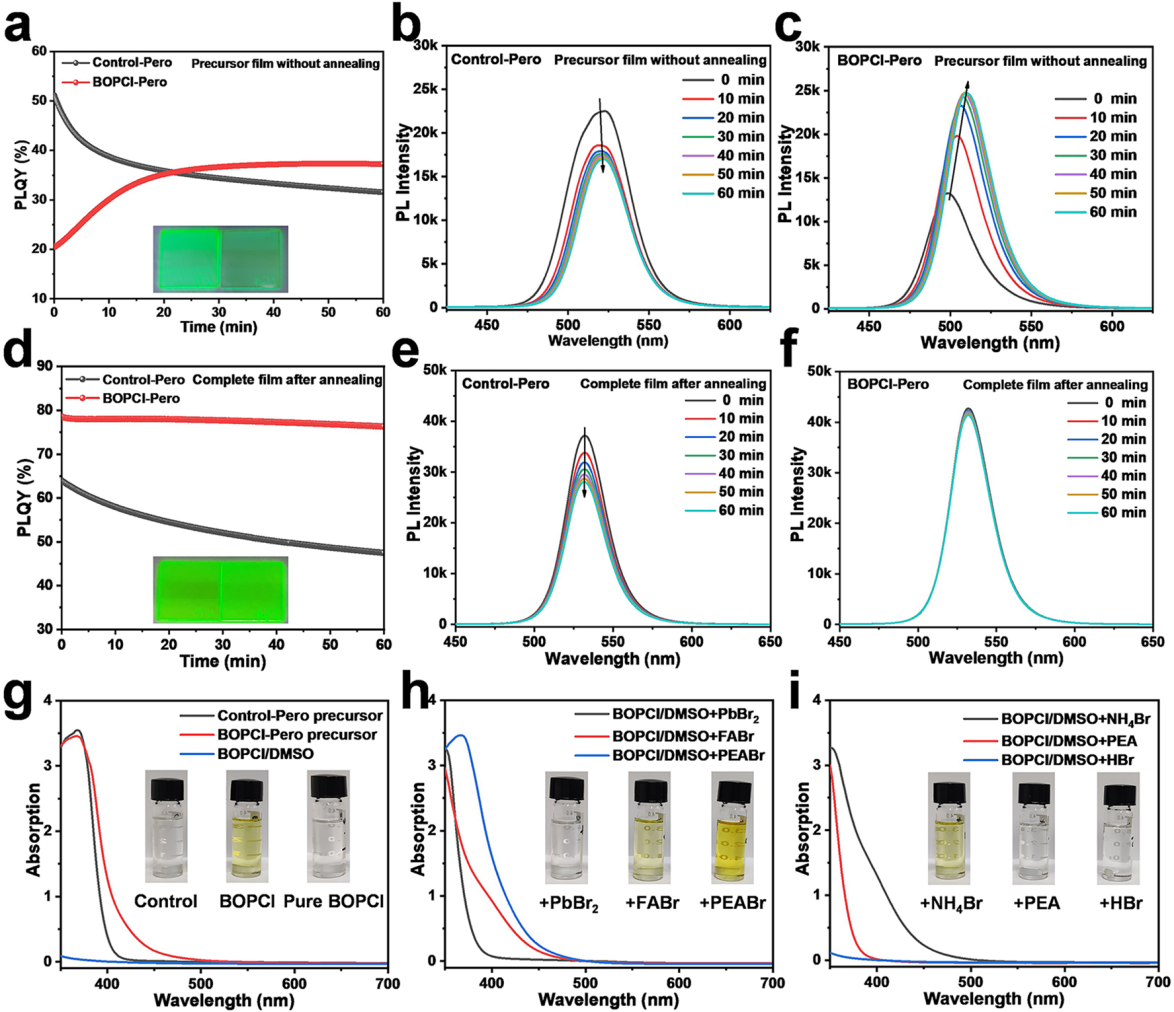
Optical property investigation of the Control-Pero and BOPCl-Pero films and precursors. **a** PLQY evolution of the precursor films without annealing and the corresponding PL spectra of **b** the Control-Pero and **c** the BOPCl-Pero films. **d** PLQY evolution of the complete films after annealing and the corresponding PL spectra of **e** the Control-Pero and **f** the BOPCl-Pero perovskite films. The insets are photographs of the Control-Pero (left) and BOPCl-Pero (right) films under UV light. UV–Vis absorption curves of **g** the Control-Pero, BOPCl-Pero precursor and the pure BOPCl in DMSO, and pure BOPCl in DMSO with different additives: **h** PbBr_2_, FABr, PEABr and **i** NH_4_Br, PEA, HBr; the insets are the photographs of the corresponding solutions

After confirming the performance enhancement from BOPCl, we turned to understand this enhancement from the film formation process. Specifically, we prepared the perovskite precursor films through spin-coating but did not carry out the annealing process, allowing the solvent to evaporate naturally. In this way, we could evaluate the crystallization rate by monitoring their PL emission evolution. As shown in Fig. 2a, in the beginning, the Control-Pero precursor films manifested a bright PL emission with PLQY of around 50%, attesting a rapid and completed crystallization process. Then, their PLQY started to decline over time, possibly due to the formation of non-radiative defects. The corresponding PL spectra are displayed in Fig. 2b. By contrast, the freshly prepared BOPCl-Pero precursor films showed a weak PL emission with PLQY of around 20%, presenting a slow and uncompleted crystallization process. Moreover, their PLQY increased significantly over time, possibly due to the following grain growth and defect elimination. And the corresponding PL spectra changes are demonstrated in Fig. 2c. These results certified that adding BOPCl could significantly slow down the crystallization process.

We also prepared the complete perovskite films with the annealing process and monitored their PL emission evaluation. As exhibited in Fig. 2d, the BOPCl-Pero films demonstrated a high and stable PLQY (~ 80%) over time. By contrast, the Control-Pero films showed a relatively lower and decreasing PLQY. The corresponding PL spectra are displayed in Fig. 2e–f. Therefore, we suspect that BOPCl may remain in the as-formed films, passivate the defects and improve the stability of perovskite film. Similar changes to BOPCl were also observed in the evolution of PLQY in BDPCl, DClP, DOPCl and DPCl-Pero precursor films without annealing and complete films after annealing (Figs. S6–S9), indicating that these phosphoryl chloride molecules have similar modification effects.

In order to understand the mechanism of crystallization retarding and defect passivation by BOPCl, we further investigated the perovskite precursor chemistry. Firstly, we found that the Control-Pero precursor and pure BOPCl/DMSO solution were colorless and transparent. In contrast, the BOPCl-Pero precursor presented a transparent yellow color, as shown in the insets of Fig. 2g. Their corresponding UV–Vis absorption spectra were collected and are plotted in Fig. 2g. The results demonstrated that, compared with the Control-Pero precursor and the pure BOPCl in DMSO, the BOPCl-Pero precursor had a new absorption at the blue wavelength of 400–500 nm, which was the reason for the yellow color of the solution, indicating an interaction happened between the BOPCl and the perovskite components. In addition, similar color changes were observed in the precursor containing other phosphoryl chloride molecules, as shown in Fig. S10, indicating that the phosphoryl chloride functional groups should play crucial roles. To further identify which component interacts explicitly with the phosphoryl chloride functional groups, PbBr_2_, FABr and PEABr were added to the BOPCl/DMSO solution, respectively. As plotted in Fig. 2h, the solution containing FABr and PEABr illustrated evident absorption at 400–500 nm, and the latter sample’s absorption was stronger, indicating that the FABr and PEABr interacted with the phosphoryl chloride functional groups, and the PEABr acted more strongly. Moreover, we found that their co-existing acting groups were positively charged ammonium, amino group containing lone pair electrons and free hydrogen bromide molecules. Therefore, corresponding substitutes were selected to be added into BOPCl/DMSO solution, as shown in Fig. 2i, for ammonium bromide (NH_4_Br) to replace ammonium, phenethylamine (PEA) to replace amino group containing lone pair electrons and hydrobromic acid (HBr, 48 wt% in H_2_O) to replace free hydrogen bromide molecules. The results proved that the solution with only NH_4_Br added had evident absorption at 400–500 nm, indicating that ammonium ions were at work. In summary, the BOPCl regulates the crystallization of perovskite film by controlling the concentration and diffusion rate of these organic cations in the precursor through the interaction of phosphoryl chloride functional groups with FA^+^ and PEA^+^ (mainly PEA^+^) [11].

### Films Properties

**Fig. 3:**
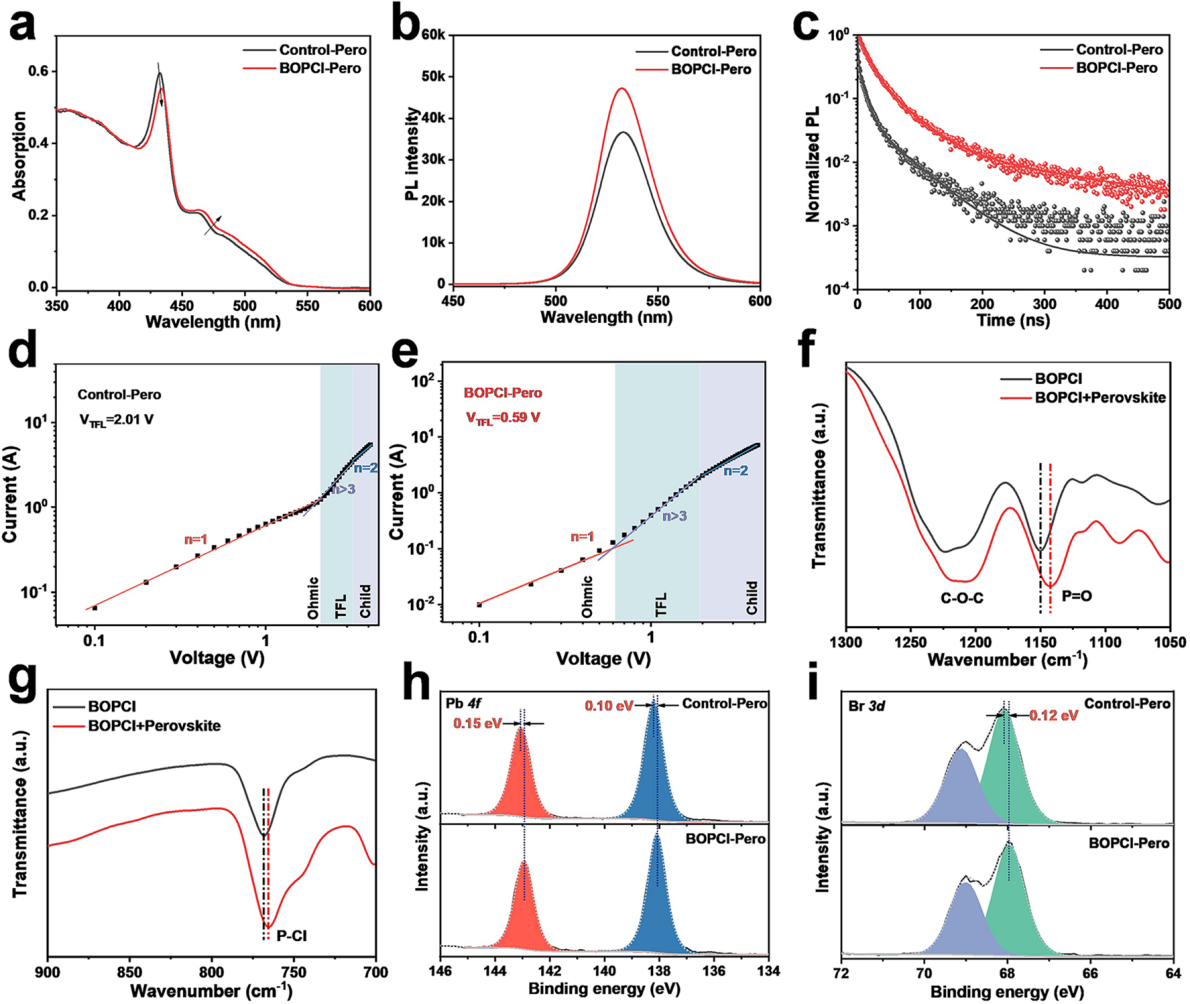
**a** UV–Vis absorption, **b** steady-state PL and **c** time-resolved PL decay curves of the Control-Pero and BOPCl-Pero films. Current–voltage curves of **d** Control-Pero film and **e** BOPCl-Pero film in SCLC measurement. FTIR spectra of the **f** P = O and **g** P-Cl functional groups in BOPCl and BOPCl-Pero films. XPS curves of the **h** Pb *4f* and **i** Br *3d* core levels of the Control-Pero and BOPCl-Pero films

To further understand the effect of the BOPCl modification on the final perovskite film, the annealed and complete Control-Pero and BOPCl-Pero films were characterized in detail. As shown in Fig. 3a, UV–Vis absorption spectroscopy measurement was used to study light absorption and phase distribution. The UV–Vis absorption curves showed that, compared to the Control-Pero film, the absorptions of low-*n* phases (~ 433 nm) in the BOPCl-Pero film were significantly reduced, while the high-*n* phases were significantly enhanced, which supported that the BOPCl could inhibit the growth of low-*n* phases and promote the growth of high-*n* phases. Further, as shown in Fig. 3b, the PL intensity of the BOPCl-Pero film was significantly stronger than the Control-Pero. Moreover, a comparison of PLQY_max_ statistics between the two perovskite films was collected and is shown in Fig. S11, and the BOPCl-Pero films showed higher values of around 80%. The results of PL and PLQY indicated the ability to passivate defects of BOPCl, in good consistent with the results in Fig. 2d. TRPL decay curves showed similar results (Fig. 3c), and the fitting details for the TRPL are summarized in Table S1. Then, the space charge limited current (SCLC) measurement, with a dark current–voltage (*I–V*) curves of typical hole-only devices (ITO/NiMgLiO_x_/Perovskite/Au), was performed to compare the defect densities of the two perovskite films quantitatively. The defect densities were calculated according to the equation of *N*_t_ = (2*εε*_0_*V*_TFL_)/(*eL*^2^), where ε is the dielectric constant of the perovskite film, *ε*_0_ is the vacuum dielectric constant, *V*_TFL_ is the trap-filled limit voltage obtained by fitting dark *I–V* curves, *e* is the elementary charge, and *L* is the thickness of the perovskite film. As shown in Fig. 3d–e, the *V*_TFL_ of Control-Pero and BOPCl-Pero films was 2.01 and 0.59 V, respectively, proving that the defect density of perovskite film was significantly reduced after the modification by BOPCl [21].

The film morphologies were characterized by SEM and AFM. As shown in Fig. S12, both films showed compact and uniform wrinkled structures, and the BOPCl-Pero film showed lower roughness (Fig. S13). And the root-mean-square roughness (*R*_q_) of the perovskite films was reduced from 17.0 nm (Control-Pero film) to 14.3 nm (BOPCl-Pero film).

FTIR and XPS were used to further investigate the interaction between the phosphoryl chloride functional groups and perovskite components. The samples used for FTIR measurement were prepared by depositing the precursor directly on the calcium fluoride substrates, including the pure BOPCl and BOPCl-Pero films. Figure S14 shows the full FTIR spectra ranging from 650 to 3700 cm^−1^ of the BOPCl and BOPCl-Pero films, and the amplified spectrum of P=O and P–Cl functional groups is shown in Fig. 3f–g. The vibration peaks of the P =O and P–Cl functional groups in BOPCl-Pero films shifted to a lower wavenumber, demonstrating that the cations did interact with the phosphoryl chloride functional groups. Figure 3h–i shows that the characteristic XPS peaks of Pb *4f* and Br *3d* in BOPCl-Pero film were shifted to lower binding energy, proving that the P=O and P–Cl functional groups (mainly P=O) donate their lone electron pair on the oxygen and chlorine atoms to the empty *6p* orbital of Pb^2+^, leading the changes in the electron cloud density of Pb^2+^ and Br^−^ [11, 22].

**Fig. 4:**
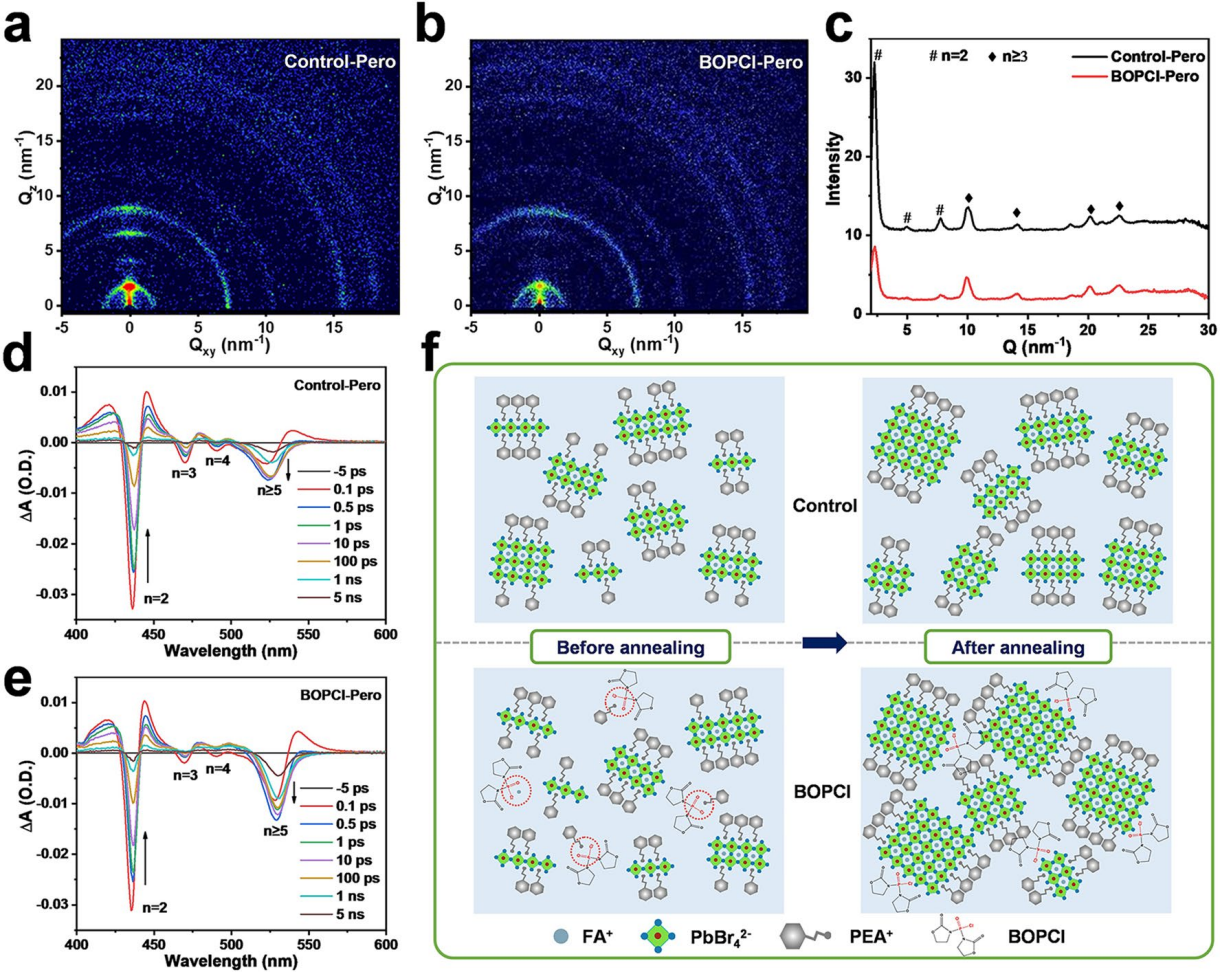
GIWAXS patterns of **a** Control-Pero and **b** BOPCl-Pero films. **c** Diffraction intensity-Q through integrating the intensity of Debye rings from GIWAXS data of Control-Pero and BOPCl-Pero films. TA spectra at a different probe delay time of **d** Control-Pero and **e** BOPCl-Pero films. **f** Schematic diagram of the impacts of BOPCl on the nucleation and growth of quasi-2D perovskites

To further investigate the phase distribution and crystallinity of the as-formed quasi-2D perovskite films, GIWAXS and XRD measurements were further performed. The GIWAXS results, as shown in Fig. 4a–c, indicated that the Control-Pero film showed well-order structures, with the quasi-2D phases oriented predominantly parallel to the substrate, similar to the previous reports [11]. By contrast, the BOPCl-Pero film showed isotropic rings, suggesting the isotropic orientation of perovskite film. The integrated intensity of diffraction rings versus Q is plotted in Fig. 4c, and diffraction peaks presented at *Q* ~ 2.3, 5 and 7.7 nm^−1^ in the Control-Pero film were significantly diminished in the BOPCl-Pero film, indicating that the formation of the low-*n* phases was suppressed by the BOPCl treatment [10]. The results of XRD measurements were consistent with the GIWAXS measurements (Fig. S15). The low-*n* phases, in the Control-Pero film, were significantly weakened or even disappeared in the BOPCl-Pero film. Meanwhile, the corresponding peaks in the quasi-2D perovskite films of the two samples had no shift, which means that the BOPCl did not enter the perovskite lattice and remained in the grain boundary.

TA measurement was performed to explore phase distribution and energy transfer dynamics. Four distinct ground-state bleaching (GSB) peaks belonging to *n* = 2, 3, 4 and *n* ≥ 5 phases were observed in the TA spectra (Fig. 4d–e) [23]. The variation of GSB peak intensity between the quasi-2D perovskite films with/without BOPCl treatment in 1 ps corresponded to the corresponding* n*-phases. And the BOPCl-Pero film had a weaker GSB peak at *n* = 2 and a stronger GSB peak at *n* ≥ 5 at 1 ps than the Control-Pero film, which proved that the treatment of BOPCl could inhibit the formation of low-*n* phases and promote the growth of high-*n* phases. Meanwhile, the GSB peaks in 1 ps mainly appeared at *n* = 2 phases, indicating that excitons are mainly produced at *n* = 2 phases [24]. The existence of the middle-*n* phases mainly plays the role of energy transfer, and the intensity evolution of the GSB peaks relates to the energy transfer in these phases. Within the first 10 ps, a rapid energy transfer could be observed, confirmed by the decreased GSB peaks of low-*n* phases along with increased GSB peaks intensity of high-*n* phases. Simultaneously, the rapid energy transfer was also demonstrated by the redshift of GSB peaks at *n* ≥ 5 phases in this stage.

### Mechanism Interpretation

Based on the above discussion, the modification mechanism of BOPCl could be concluded and is schematically shown in Fig. 4f. The improvement of BOPCl modification to the quasi-2D Pero-LEDs is mainly due to the improved performance of the perovskite films, and BOPCl has a dual role of regulating crystallization and passivating defects. Firstly, the BOPCl can limit the diffusion of the organic cations (PEA^+^ and FA^+^) in the initial film-forming process by the interaction between the phosphoryl chloride functional groups and these organic cations, thereby slowing down the crystallization and suppressing the formation of low-*n* phases. Then, the remaining BOPCl in the perovskite films would interact with the uncoordinated cations (mainly Pb^2+^) and play a passivation role.

## Conclusion

In summary, we found through the molecular screening that a series of molecules containing phosphoryl chloride functional groups have obvious improvement effects on quasi-2D Pero-LEDs. Then, we investigated the modification mechanism by focusing on the BOPCl. It is found that the BOPCl can delay the crystallization rate, thus inhibiting the excess formation of low-*n* phases and promoting the growth of high-*n* phases. Meanwhile, the remaining BOPCl in the quasi-2D perovskite film would interact with the uncoordinated cations and passivate the non-radiative defects. In this way, taking advantage of the crystallization regulation and defect passivation provided by phosphoryl chloride functional groups, a quasi-2D green Pero-LEDs with an EQE_max_ of 20.82% and an EQE_ave_ of about 20% on 50 devices were obtained. Good reproducibility was demonstrated. There is no denying that our work provides useful ideas for the development of quasi-2D Pero-LEDs to achieve high performance and reproducibility.

### Supplementary Information

Below is the link to the electronic supplementary material.

Supplementary file1 (PDF 1685 kb)
