# Supplementary material for: Phase Regulation and Defect Passivation Enabled by Phosphoryl Chloride Molecules for Efficient Quasi-2D Perovskite Light-Emitting Diodes
Source: Nanomicro Lett. 2023 May 1;15:119. doi: 10.1007/s40820-023-01089-3 (PMC10151432; doi:10.1007/s40820-023-01089-3)
Supplement: Supplementary file 1 — Supplementary file1 (PDF 1685 kb) [file 40820_2023_1089_MOESM1_ESM.pdf]

Supporting Information for

Phase Regulation and Defect Passivation Enabled by Phosphoryl Chloride Molecules for Efficient Quasi-2D Perovskite Light-Emitting Diodes

Mingliang Li[1, 2, †], Yaping Zhao[2, †], Jia Guo[3, *], Xiangqian Qin[2], Qin Zhang[2], Chengbo Tian[2], Peng Xu[2], Yuqing Li[2], Wanjia Tian[2], Xiaojia Zheng[1], Guichuan Xing[3], Wen-Hua Zhang[1, 5, *], and Zhanhua Wei[2, 4, *]

[1]Sichuan Research Center of New Materials, Institute of Chemical Materials, China Academy of Engineering Physics, Chengdu 610200, P. R. China

[2]Xiamen Key Laboratory of Optoelectronic Materials and Advanced Manufacturing, Institute of Luminescent Materials and Information Displays, College of Materials Science and Engineering, Huaqiao University, Xiamen 361021, P. R. China

[3]Joint Key Laboratory of the Ministry of Education, Institute of Applied Physics and Materials Engineering, University of Macau, Taipa, Macau 999078, P. R. China

[4]Innovation Laboratory for Sciences and Technologies of Energy Materials of Fujian Province (IKKEM), Xiamen 361005, P. R. China

[5]School of Materials and Energy, Yunnan University, Kunming 650050, P. R. China

†Mingliang Li and Yaping Zhao contributed equally to this work.

*Corresponding authors. E-mails: jiaguo@um.edu.mo (Jia Guo); wenhuazhang@ynu.edu.cn (Wen-Hua Zhang); weizhanhua@hqu.edu.cn (Zhanhua Wei)

Supplementary Figures and Table

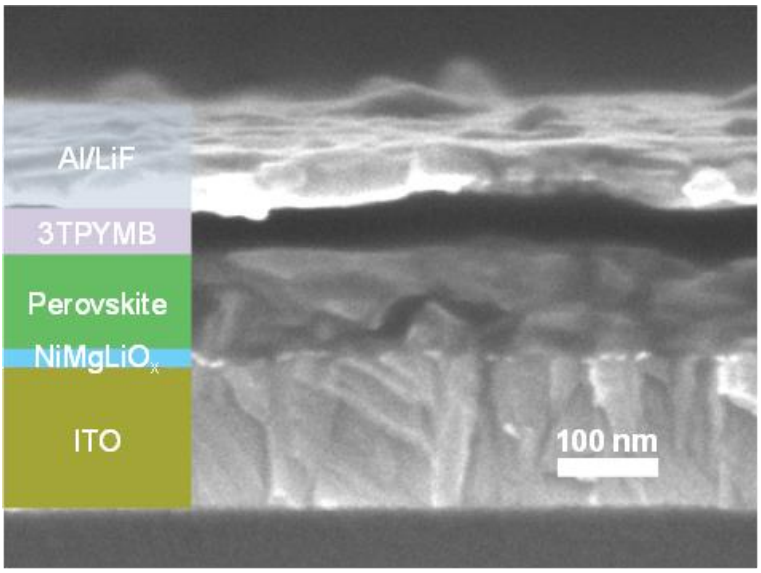

Fig. S1 The cross-sectional SEM image of the Pero-LEDs based on BOPCl-Pero film

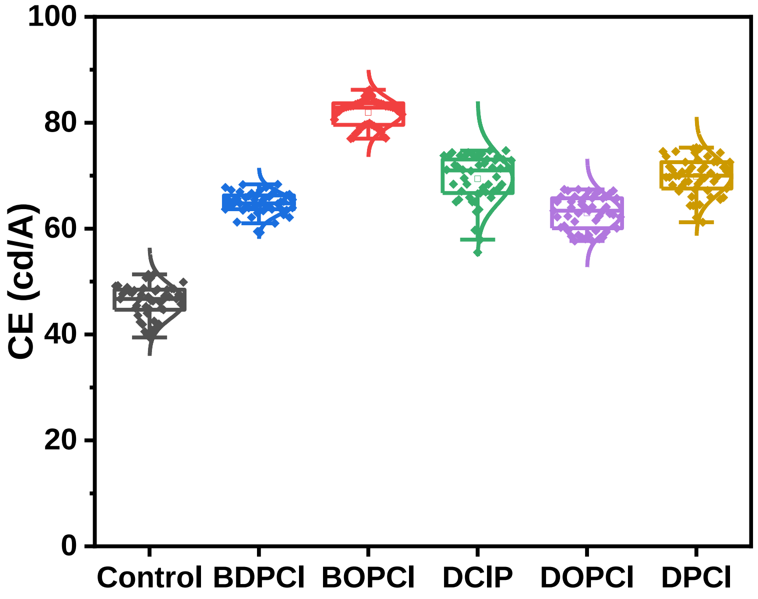

Fig. S2 CE_{max} histogram of the best 50 Pero-LEDs fabricated with Control-Pero films and other perovskite films modified by various phosphoryl chloride molecules

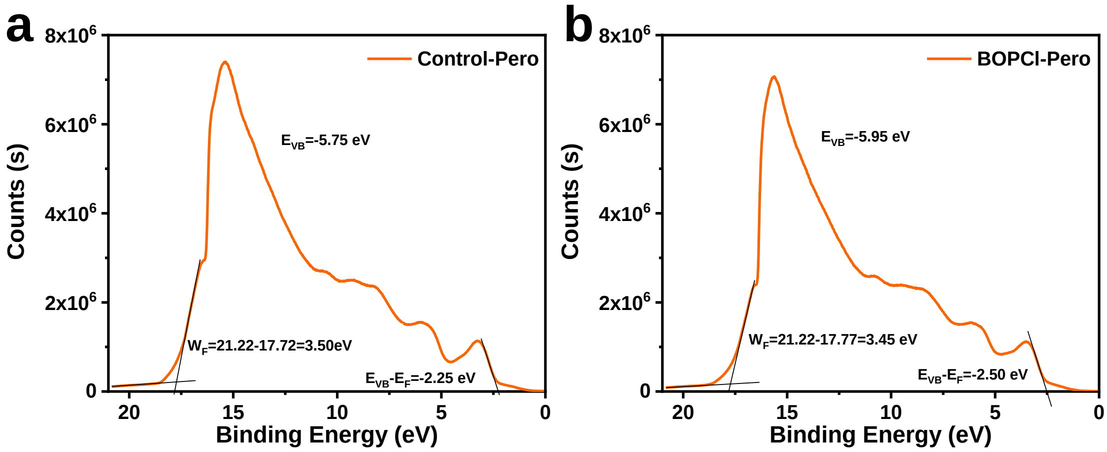

Fig. S3 UPS date of the (**a**) Control-Pero and (**b**) BOPCl-Pero films

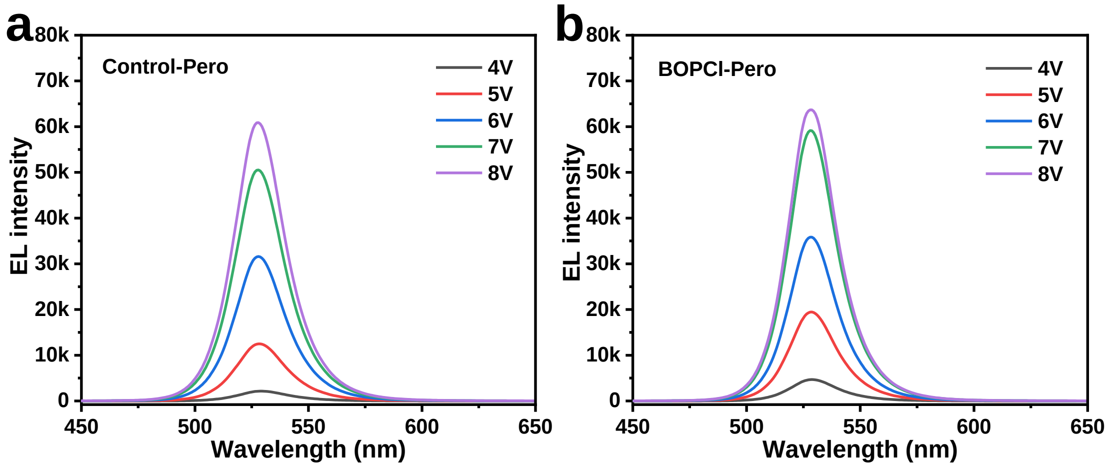

Fig. S4 EL spectra of the Pero-LEDs based on (**a**) Control-Pero and (**b**) BOPCl-Pero films

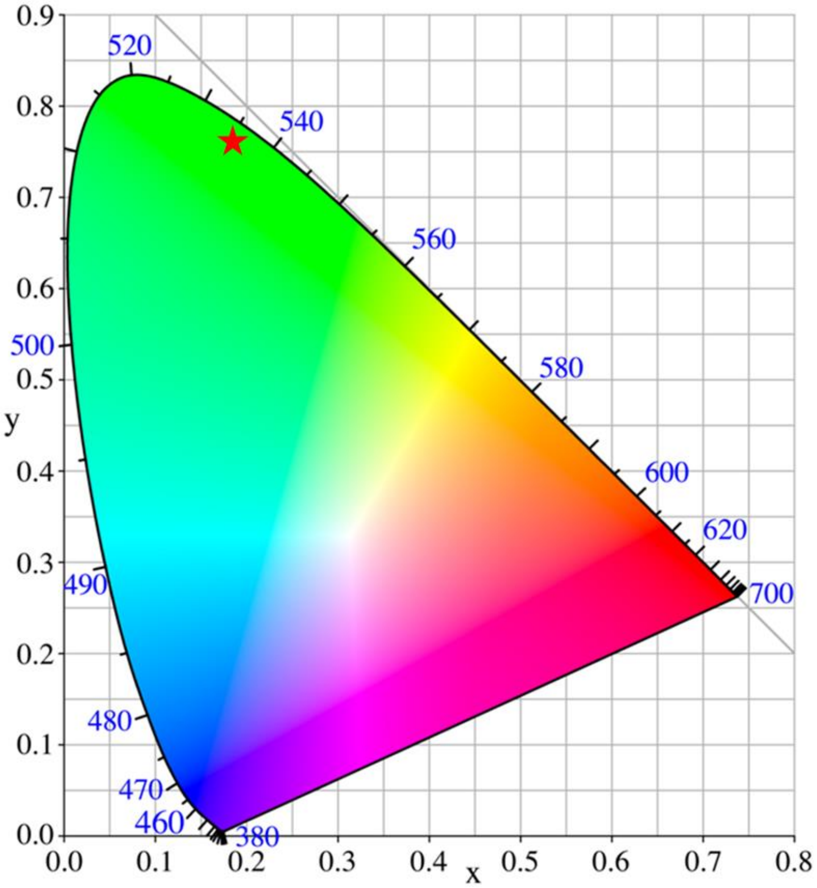

Fig. S5 CIE coordinate of Pero-LEDs based on BOPCl-Pero film

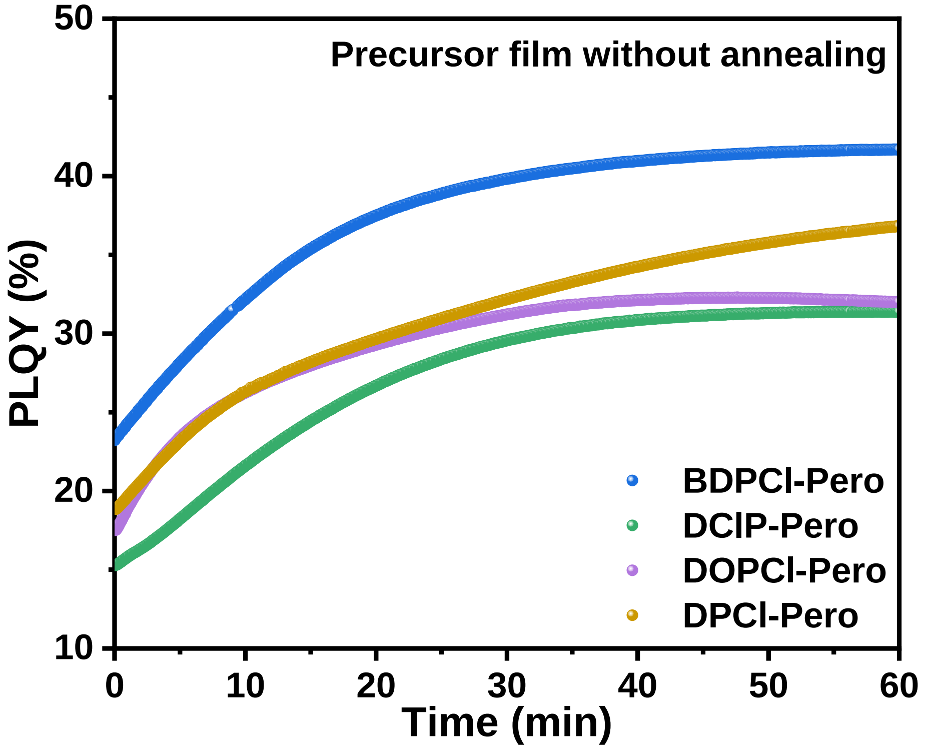

Fig. S6 PLQY evolution of the BDPCl, DClP, DOPCl and DPCl-Pero precursor films without annealing

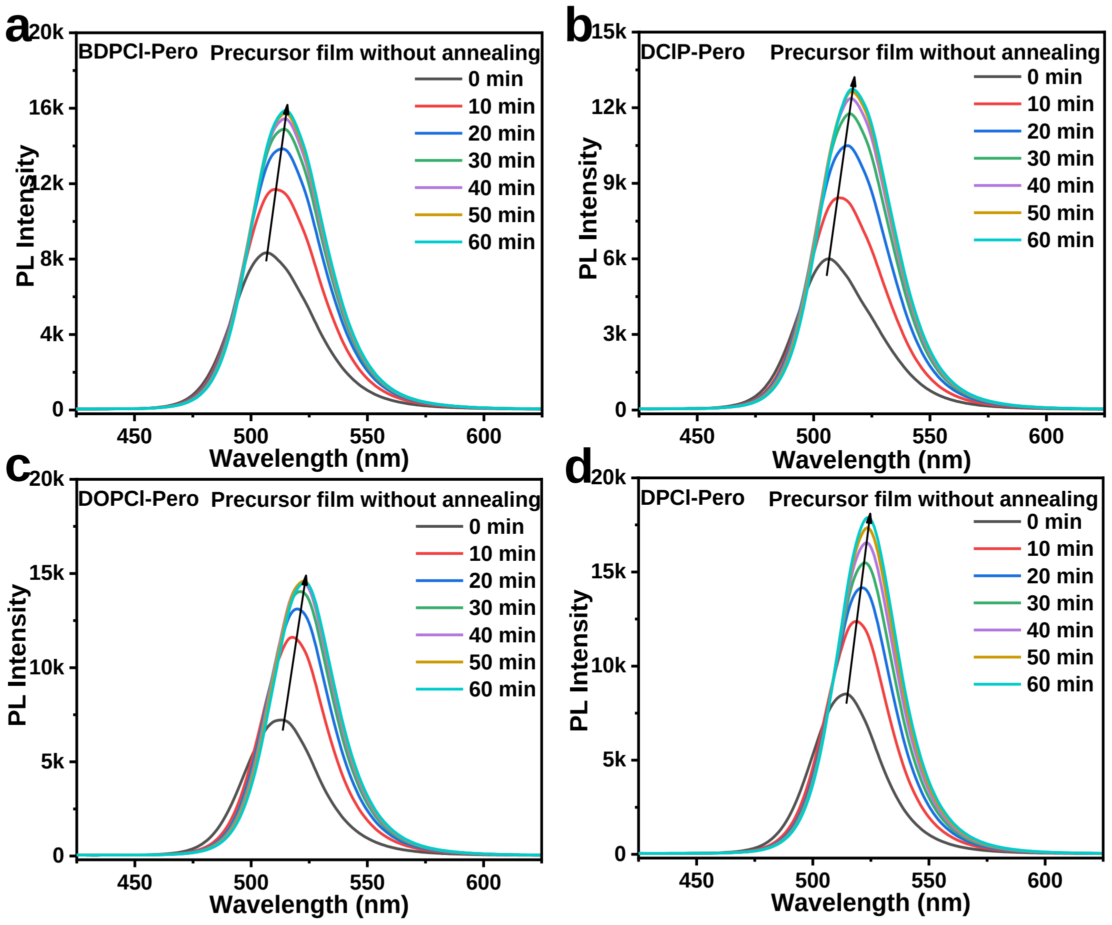

Fig. S7 The corresponding PL spectra for the PLQY evolution of the BDPCl, DClP, DOPCl and DPCl-Pero precursor films without annealing

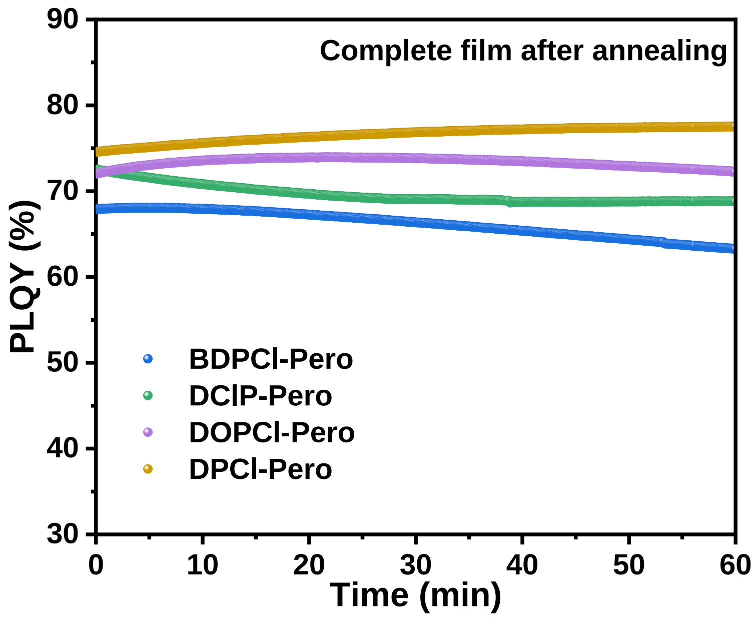

Fig. S8 PLQY evolution of the BDPCl, DClP, DOPCl and DPCl-Pero complete films after annealing

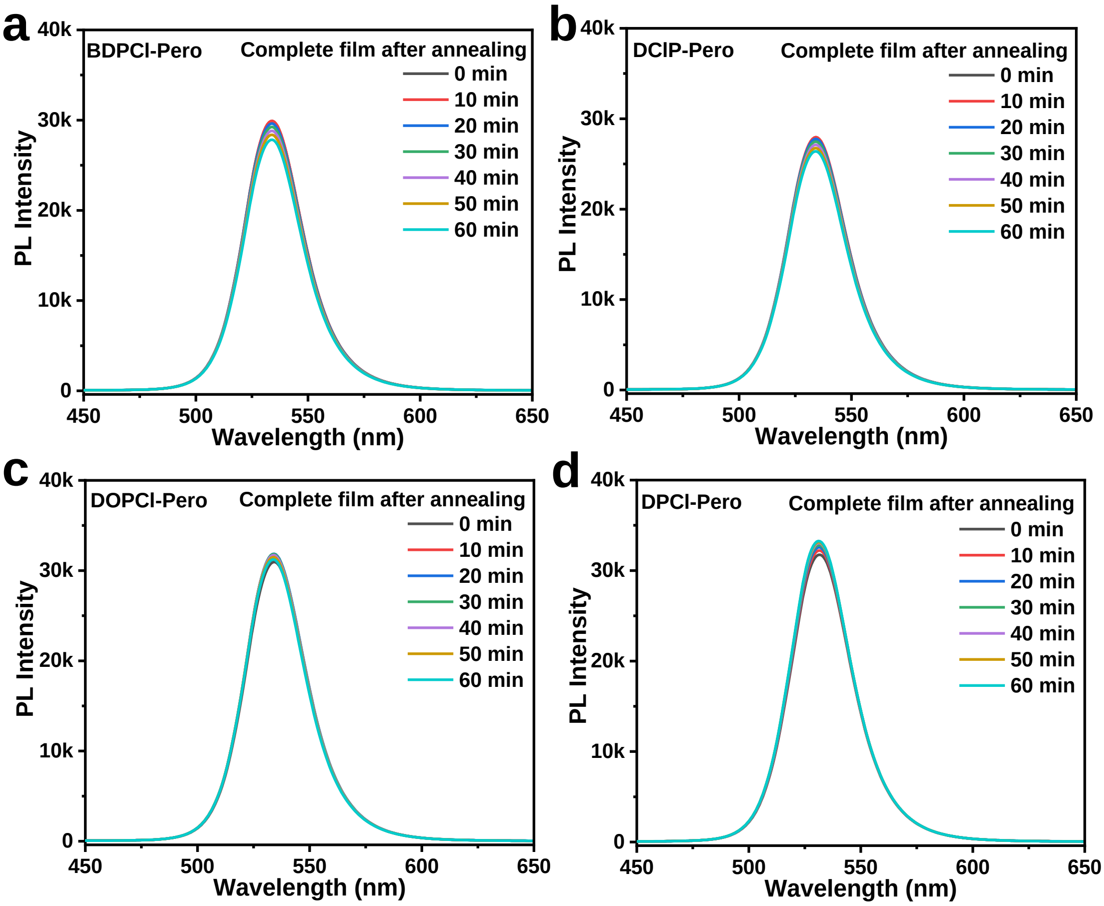

Fig. S9 The corresponding PL spectra for the PLQY evolution of the BDPCl, DClP, DOPCl and DPCl-Pero complete films after annealing

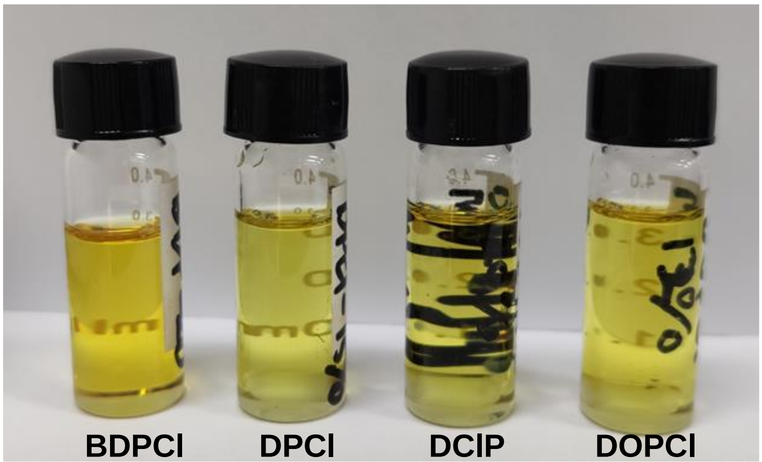

Fig. S10 Photographs of perovskite precursor with BDPCl, DPCl, DClP and DOPCl

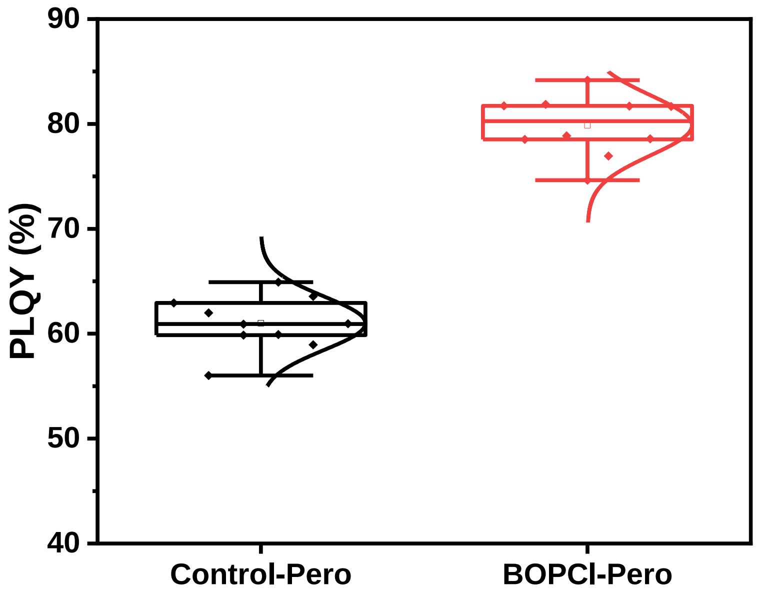

Fig. S11 PLQY histogram of the 10 films of Control-Pero and BOPCl-Pero films

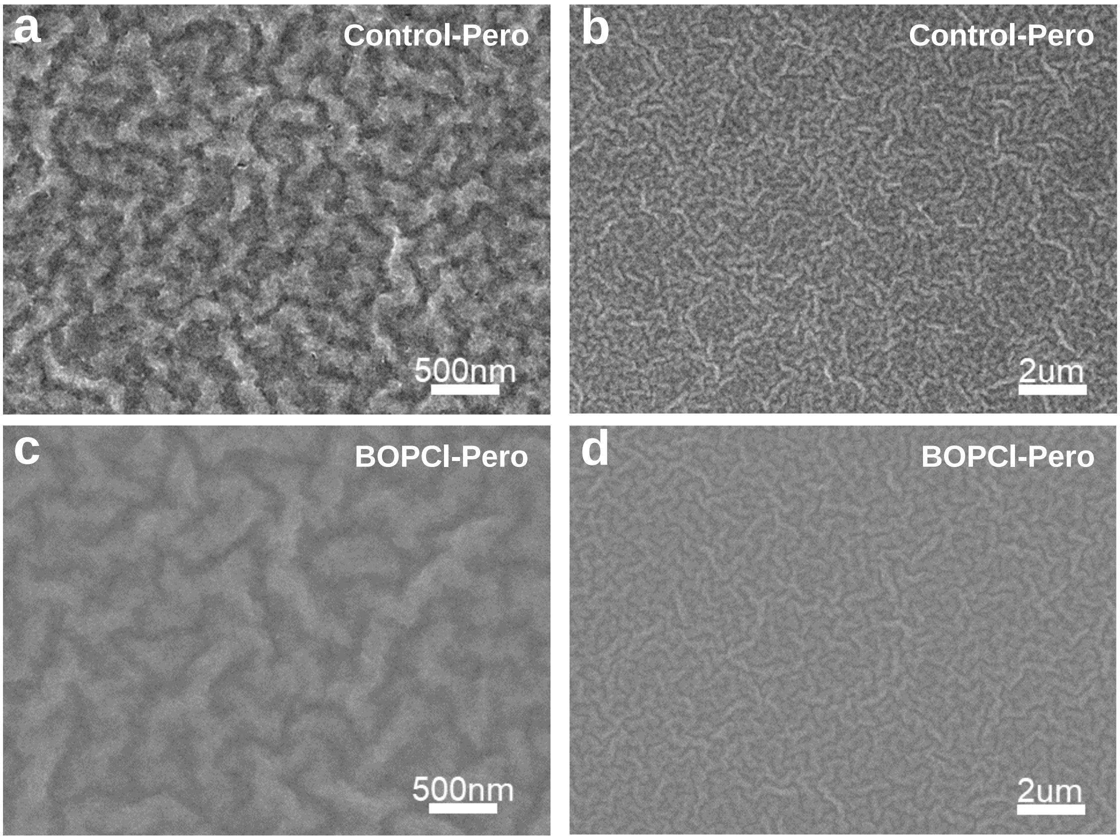

Fig. S12 SEM images of (**a, b**) Control-Pero and (**c, d**) BOPCl-Pero films

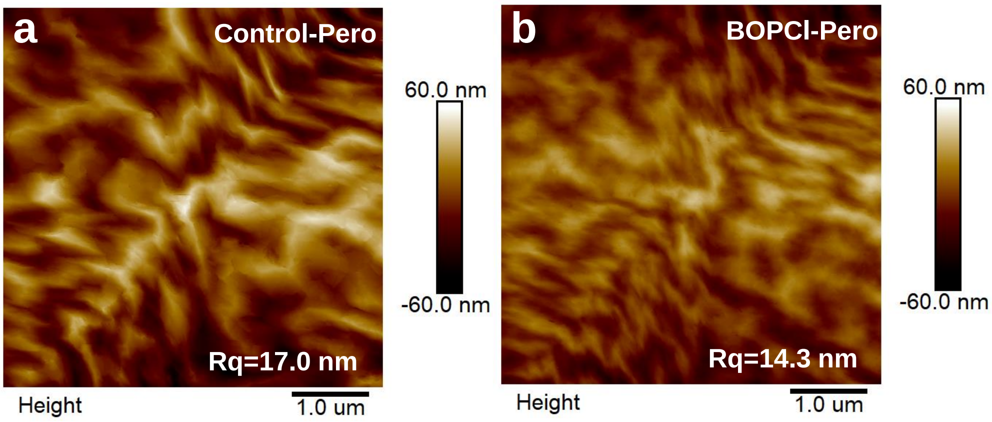

Fig. S13 AFM images of (**a**) Control-Pero and (**b**) BOPCl-Pero films

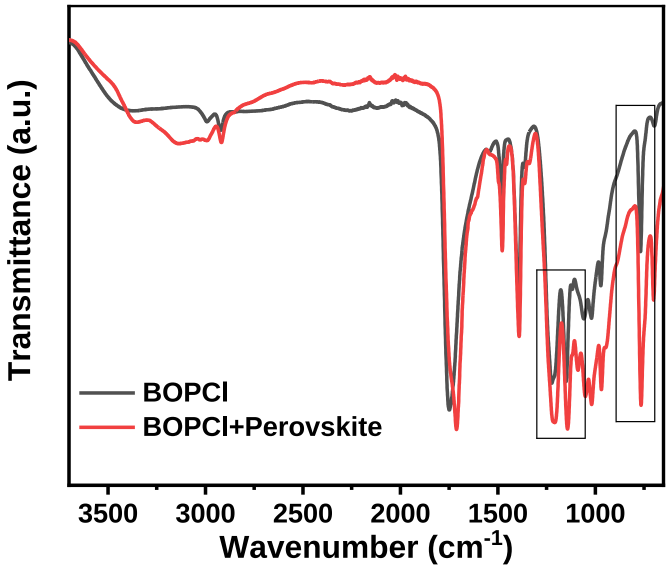

Fig. S14 The full FTIR spectra ranging from 650 to 3700 cm^{-1} of the BOPCl and BOPCl-Pero films

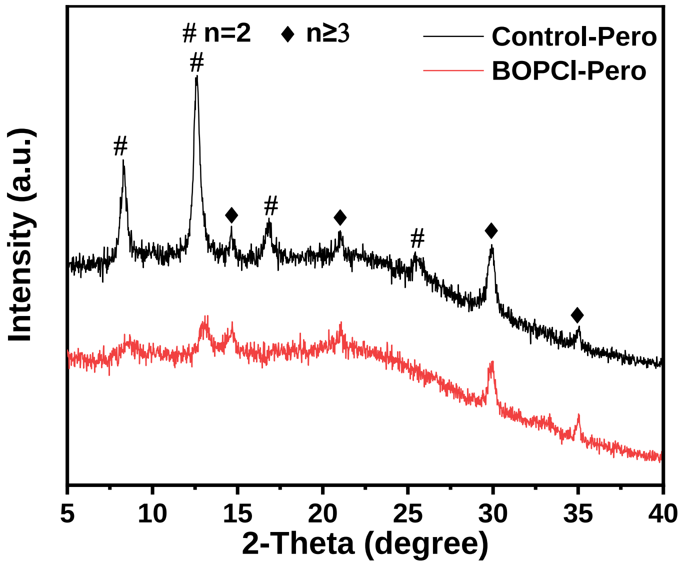

Fig. S15 XRD patterns of the Control-Pero and BOPCl-Pero films

Table S1 Fitting results of the TRPL decay curves of the Control-Pero and BOPCl-Pero films

	τ_1 (ns)	A_1	τ_2 (ns)	A_2	τ_3 (ns)	A_3	χ^2 (%)	τ_{ave} (ns)
Control-Pero	10.17	0.28	1.02	0.66	53.4	0.06	99.9	29.85
BOPCl-Pero	13.47	0.63	41.48	0.35	242.06	0.02	99.9	67.81
